# Robust Markers Reflecting Phylogeny and Taxonomy of Rhizobia

**DOI:** 10.1371/journal.pone.0044936

**Published:** 2012-09-17

**Authors:** Yan Ming Zhang, Chang Fu Tian, Xin Hua Sui, Wen Feng Chen, Wen Xin Chen

**Affiliations:** 1 State Key Laboratory of Agrobiotechnology, College of Biological Sciences, China Agricultural University, Beijing, China; 2 Key Laboratory of Soil Microbiology, Ministry of Agriculture, China Agricultural University, Beijing, China; 3 Rhizobium Research Center, China Agricultural University, Beijing, China; J. Craig Venter Institute, United States of America

## Abstract

Genomic ANI (Average Nucleotide Identity) has been found to be able to replace DNA-DNA hybridization in prokaryote taxonomy. The ANI of each of the core genes that has a phylogeny congruent with the reference species tree of rhizobia was compared to the genomic ANI. This allowed us to identify three housekeeping genes (*SMc00019*-*truA*-*thrA*) whose ANI reflected the intraspecies and interspecies genomic ANI among rhizobial strains, revealing an ANI gap (≥2%) between the inter- and intra-species comparisons. The intraspecies (96%) and interspecies (94%) ANI boundaries calculated from three genes (*SMc00019*-*truA*-*thrA*) provided a criterion for bacterial species definition and confirmed 621/629 of known interspecies relationships within *Bradyrhizobium*, *Mesorhizobium*, *Sinorhizobium* and *Rhizobium*. Some widely studied strains should be renamed. The *SMc00019*-*truA*-*thrA* ANI also correlates well with the genomic ANI of strains in *Agrobacterium*, *Methylobacterium*, *Ralstonia*, *Rhodopseudomonas*, *Cupriavidus* and *Burkholderia*, suggesting their wide applicability in other bacteria.

## Introduction

Theory-based concepts of prokaryotic species have been debated in the community [1] but, pragmatically, prokaryotic species have been defined by polyphasic approaches including phenotypic and genetic methods. Among these, the 70% DDH (DNA-DNA hybridization) value has been widely used as a gold standard, especially where 16S rRNA gene sequence similarity values are more than 97% [2]. However, DDH has serious limitations: it is time-consuming, ill-suited for rapid identification, and unavailable for non-culturable prokaryotes. Moreover the 70% DDH criterion does not correspond to an ecological/evolutionary theory-based concept of what properties a species should have [3]. The advance of sequencing technology has allowed us to use comparative genomics and multi-locus sequence analysis (MLSA) to provide a closer marriage between the definition and concept of species [3]. Recently, 95–96% ANI (Average Nucleotide Identity) of pairwise genomes has been demonstrated to correlate with the current boundary of the 70% DDH value [4]–[6]. In a phylogenetic and/or taxonomic survey, however, it is unnecessary to sequence genomes for all the prokaryotes under study and genome assembly may not even be possible, as in metagenomics. Thus, efforts have been made either to reduce the sequencing coverage of genomes or to screen fewer loci while reflecting the average signatures of whole genomes [4], [7]. Up to now, MLSA has been widely used to infer the taxonomy, phylogeny and microevolution of prokaryotes [2], [3], but a universal set of genes for all prokaryotes might be unattainable because those ubiquitous genes conserved enough to be amplified by general primers might not evolve quickly enough to distinguish closely related taxa [3]. Moreover, the species- and subspecies- level analyses dominated the topics of most studies and are increasingly important in microbiome survey [8], [9].

Rhizobia are defined as symbiotic bacteria forming nitrogen-fixing nodules with diverse legumes, and they play an important role in sustainable agriculture. More than 90 species belonging to 13 genera of α- and β- proteobacteria have been described as rhizobia. Due to the limited number of rhizobial genomes in databases, target genes (*atpD*, *dnaK*, *gap*, *glnA*, *glnII*, *gltA*, *gyrB*, *pnp*, *recA*, *rpoB* and *thrC*) for MLSA of rhizobia have been selected largely in the belief that they were housekeeping genes [10]–[12]. No comparisons between the phylogenies of these genes and phylogenies derived from whole genome data were made before using them. Although different subsets of these possible housekeeping genes were carefully chosen for further analyses in each genus, incongruent phylogenetic signals among these loci have been reported [10]–[12]. Moreover, for these possible housekeeping loci, a universal clear-cut gap between intra- and inter-specific sequences was not found [10]–[12]. A recent comparative genomic study of 22 rhizobial genomes identified 33 core genes with phylogenies congruent with the reference tree based on 295 core genes [13]. In the present study, we first tested whether the ANI of each of these 33 genes reflected the ANI of the corresponding pair of genomes. The best performing markers were then used to evaluate the current taxonomy of type strains in *Bradyrhizobium*, *Mesorhizobium*, *Sinorhizobium* (later synonym *Ensifer*; due to more extensive use of *Sinorhizobium* in the community, *Sinorhizobium* was used throughout the text), and *Rhizobium*. The correlation between the ANI of selected markers and the genomic ANI of published genomes in *Agrobacterium*, *Methylobacterium*, *Ralstonia*, *Rhodopseudomonas*, *Cupriavidus* and *Burkholderia* was also studied.

## Materials and Methods

### Genomes and housekeeping genes

The 85 genomes used in this study are listed in Table S1. Thirty-three core genes with phylogenies congruent with the reference species tree of 22 rhizobial genomes were found earlier [13], but five of these genes are absent in other rhizobial genomes. Therefore, 28 out of 33 genes were analyzed herein (Table 1).

**Table 1:** 28 core genes of rhizobia used in this study.

Gene: Function	COG Category
***recR***: Recombination protein	Replication, recombination and repair
***recQ***: ATP-dependent DNA helicase	Replication, recombination and repair
***SMc00019***: Conserved hypothetical protein	Transcription
***rpoB***: DNA-directed RNA polymerase beta chain	Transcription
***rpoC***: DNA-directed RNA polymerase beta chain	Transcription
***prfC***: Peptide chain release factor RF-3	Translation, ribosomal structure and biogenesis
***leuS***: Leucyl-tRNAsynthetase	Translation, ribosomal structure and biogenesis
***truA***: RNA pseudouridine synthase A	Translation, ribosomal structure and biogenesis
***glyS***: Glycyl-tRNA synthetase beta chain	Translation, ribosomal structure and biogenesis
***rplB***: 50S ribosomal protein L2	Translation, ribosomal structure and biogenesis
***thrA***: Homoserine dehydrogenase	Amino acid transport and metabolism
***aroB***: 3-dehydroquinate synthase transmembrane protein	Amino acid transport and metabolism
***lysC***: Aspartokinase	Amino acid transport and metabolism
***dac***: D-alanyl-D-alanine carboxypeptidase fraction A	Cell wall/membrane/envelope biogenesis
***murC***: UDP-N-acetylmuramate–alanine ligase	Cell wall/membrane/envelope biogenesis
***acnA***: Aconitatehydratase	Energy production and conversion
***glpK***: Glycerol kinase	Energy production and conversion
***ctaE***: Cytochrome C oxidase subunit III transmembrane protein	Energy production and conversion
***hemF***: Coproporphyrinogen III oxidase, aerobic protein	Coenzyme transport and metabolism
***SMc01147***: Oxygen-independent coproporphyrinogen III oxidase	Coenzyme transport and metabolism
***dnaK***: Heat shock protein 70 (HSP70) chaperone	Posttranslational modification, protein turnover, chaperones
***secA***: Preprotein translocase SecA subunit	Intracellular trafficking, secretion, and vesicular transport
***glgB1***: 1,4-alpha-glucan branching enzyme protein	Carbohydrate transport and metabolism
***SMc01146***: HAM1 NTPase family protein	Nucleotide transport and metabolism
***SMc00714***: 1-acyl-SN-glycerol-3-phosphate acyltransferase	Lipid transport and metabolism
***SMc02059***: Conserved hypothetical protein	General function prediction only
***SMc02478***: Conserved hypothetical protein	General function prediction only
***cgtA***: GTP-binding protein	General function prediction only

### Average nucleotide identity (ANI) analysis

ANI between genomes (ANIm) was calculated by using the NUCmer algorithm [14] integrated in Jspecies [4].The “No. of differences” model integrated in MEGA 5 [15] was used for calculating the pairwise distance between sequences of a single gene, from which ANI values (ANIg) were obtained using Excel. Pearson and Spearman correlation coefficients between ANIg and ANIm values were calculated using SPSS. Curve estimation integrated in SPSS was used to find the curve that gives the best fit to the data of ANIm-ANIg pairs.

### Phylogenetic and recombination analysis

All of the 28 test genes passed the Permutation Tail Probability test (PTP) integrated in PAUP* 4.0b10 [16]. Single gene alignments were aligned by using MEGA 5 [15]. Modeltest [17] was used to produce the best nucleotide substitution model for each alignment. Maximum likelihood (ML) trees with 100 bootstrap replicates were constructed by PhyML 3.0 [18], or by PAUP* 4.0b10 [16] with PHYLIP [19] for bootstrapping. The Shimodaira-Hasegawa (SH) test [20] integrated in PAUP* 4.0b10 was performed to test the level of topological congruence between ML trees (*P* = 0.05). The PHI test [21] was used to detect potential recombination signal in single or concatenated genes.

### Primers and PCR conditions

Degenerate primers (Table 2) were designed for the amplification of *thrA*, *SMc00019* and *truA* in 67 type strains of *Bradyrhizobium*, *Sinorhizobium*, *Mesorhizobium* and *Rhizobium* (Table S2). Fragment amplifications were carried out by PCR with primer pairs as follows: *thrA*B-F/R (*Bradyrhizobium*), *thrA*MRS-F/R (*Mesorhizobium*, *Rhizobium* and *Sinorhizobium*), *SMc00019*B-F/R (*Bradyrhizobium*), *SMc00019*MRS-F/R (*Mesorhizobium*, *Rhizobium* and *Sinorhizobium*), *truA*B-F/R (*Bradyrhizobium*), *truA*R-F/R (*Rhizobium*) and *truA*MS-F/R (*Mesorhizobium* and *Sinorhizobium*). Corresponding PCR conditions were listed in Table 2. The resulting 201 sequences were deposited in GenBank under the accession numbers JX064199-JX064265 (*SMc00019*), JX064271-JX064337 (*truA*) and JX064343- JX064409 (*thrA*).

**Table 2:** Primers used in this study.

Primer pair: sequence (5′-3′)	Customized intermediate PCR cycling*
*thrA*B-F: TGC TTC GTC GAR YTG ATG G and *thrA*B-R: ACR CCC ATC ACC TGY GCR ATC	13×(45 s 94°C, 1 min 61°C to 48°C with −1°C/cycle, 1 min 72°C), 22×(45 s 94°C, 1 min 48°C, 1 min 72°C)
*thrA*MRS-F: GCN GGB GGY ATY CCS GTB ATC AAG and *thrA*MRS-R: CGY TCG ATN CGR ATS ACY TGS GG	10×(45 s 94°C, 1 min 66°C to 56°C with −1°C/cycle, 1 min 72°C), 25×(45 s 94°C, 1 min 56°C, 1 min 72°C)
*SMc00019*B-F: CAT TCV KCS GAR GGV GCS ATG GGY ATC and *SMc00019*B-R: GCG TGB CCB GCS KCG TTS GAV AGC AT	30×(45 s 94°C, 1 min 66°C, 1 min 72°C)
*SMc00019*MRS-F: CAD TTC CTB ATH GCC ATG CC and *SMc00019*MRS-R: GCV GGR CAN KTS AGC CAD CCR TT	15×(45 s 94°C, 1 min 66°C to 51°C with −1°C/cycle, 1 min 72°C), 20×(45 s 94°C, 1 min 50°C, 1 min 72°C)
*truA*B-F: CGC TAC AAG CTC AYY ATC GA and *truA*B-R: CCS ACC ATS GAG CGB ACC TG	10×(45 s 94°C, 1 min 60°C to 50°C with −1°C/cycle , 1 min 72°C), 25×(45 s 94°C, 1 min 50°C, 1 min 72°C)
*truA*R-F: TGA CCG TSG AAT ATG ACG G and *truA*R-R: ACA TCS AGY CGG TCV AGS GT	7×(45 s 94°C, 1 min 58°C to 51°C with −1°C/cycle, 1 min 72°C), 28×(45 s 94°C, 1 min 51°C, 1 min 72°C)
*truA*MS-F: CAG GTS GCD CAT STC GAY CT and *truA*MS-R: GAD CGB AYC TGG TTR TGM AG	10×(45 s 94°C, 1 min 58°C to 48°C with −1°C/cycle, 1 min 72°C), 25×(45 s 94°C, 1 min 48°C, 1 min 72°C)

Note: N = A, G, C or T; R = A or G; Y = C or T; M = A or C; S = G or C; K = G or T; V = A, C or G; D = A, G or T; H = A, C or T; B = C, G or T.

* , Fragment applications were carried out by PCR with an initial denaturation at 95°C for 5 min, final extension at 72°C for 10 min, and customized intermediate PCR cycles for each primer pair.

## Results and Discussion

### Novel phylogenetic and taxonomic markers and re-evaluation of current classification of Rhizobiaceae

**Figure 1:**
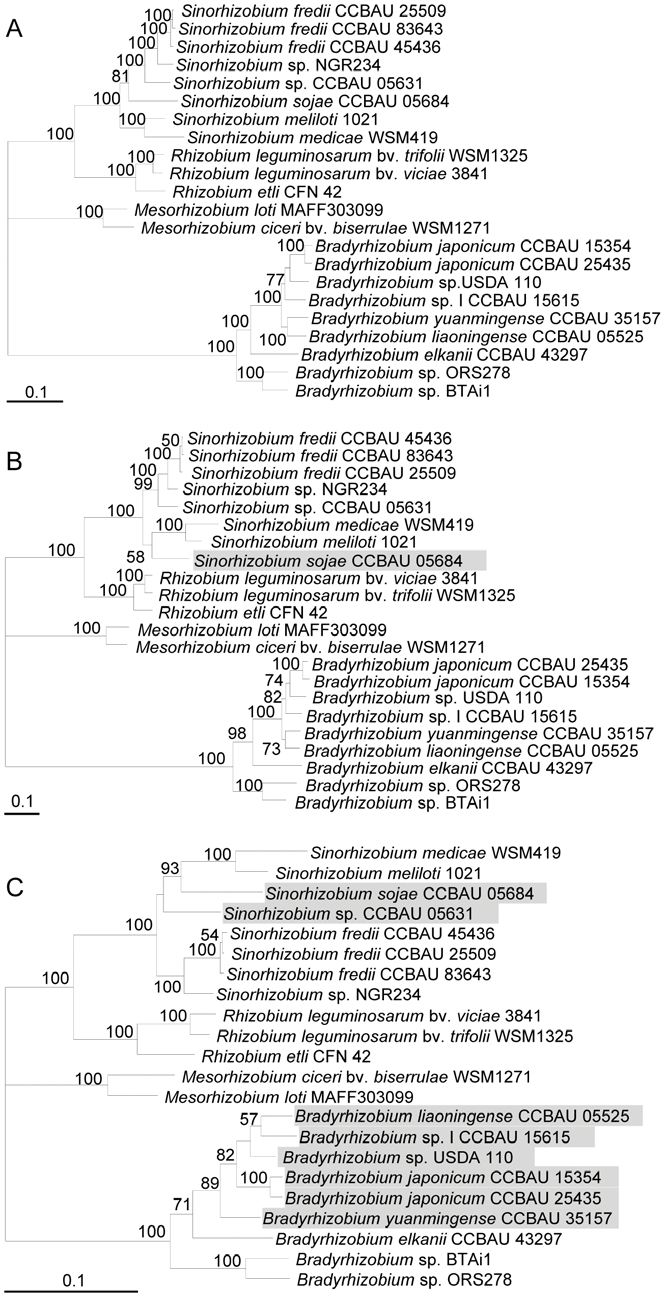
The maximum likelihood (ML) phylogenetic trees. The tree was constructed by PhyML based on concatenated sequences of 28 core genes (A), *SMc00019-truA-thrA* concatenate (B) or *recA-glnII*-*atpD* concatenate (C). Bootstrap confidence levels ≥50% are indicated at the internodes. Bar = 10% nucleotide divergence.

Consistent with the genomic ANIm 95%–96% boundary for the species definition of other prokaryotes, it was recently shown that genomic ANIm >95% could be used to identify strains of the same rhizobial species [4], [13]. Among the 295 core genes of rhizobial genomes, single-gene trees (ML) of 33 core genes have been found to have phylogenies congruent with the reference species tree based on concatenation of 295 core genes or with the strict consensus tree of 295 single-gene trees [13]. In this study, 28/33 of these core genes (Table 1) were further analyzed. An ML tree based on the concatenated sequences of these 28 genes (Figure 1A) was found to have a topology highly congruent (SH-test, *P* = 1.0) with the rhizobial species tree described earlier [13], and was used as the reference species tree herein. In order to find potential taxonomic markers among these 28 genes, we calculated their ANIg values among rhizobial strains for which genome sequences are available (Table S1). Twelve genes satisfied the criterion that ANIg 95% or 96% serves as the boundary of rhizobial species corresponding to the species assignments based on genomic ANIm values (with 95% as the boundary, see below). Among these genes, *SMc00019*, *truA* and *thrA* were selected for further analyses, considering their shorter gene length (<1.5 kb) and more similar topology to the reference tree.

The ML phylogenetic tree of concatenated *SMc00019*-*truA*-*thrA* (Figure 1B) had higher bootstrap values than the individual gene trees of *SMc00019*, *truA*, and *thrA*, and showed significant congruent topology (SH-test, P = 0.348) to the reference species tree (Figure 1A). Consequently, the *SMc00019*-*truA*-*thrA* concatenation could be used for the construction of a robust phylogenetic tree of rhizobia.

**Figure 2:**
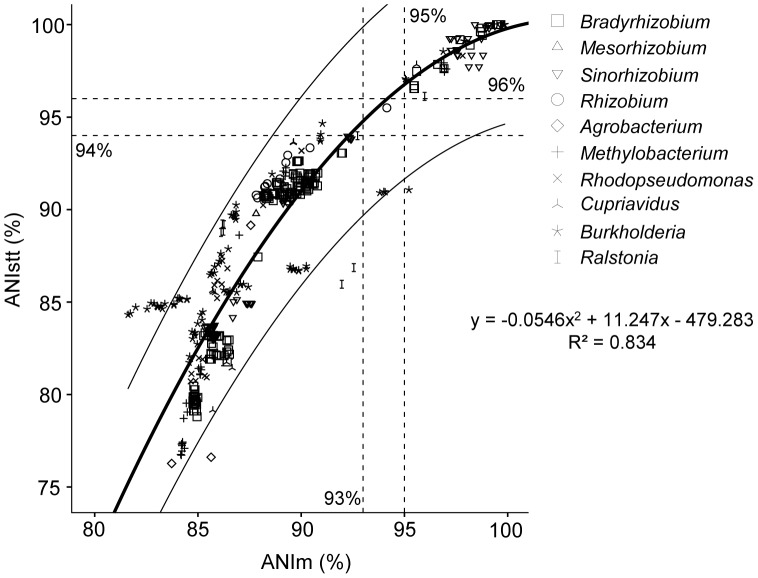
Plotted results of ANIstt (average nucleotide identity of *SMc00019-truA-thrA*) versus ANIm (genomic ANI). 460 intra-genus pairwise comparisons in *Bradyrhizobium* (190), *Mesorhizobium* (3), *Sinorhizobium* (91), *Rhizobium* (10), *Agrobacterium* (6), *Cupriavidus* (10), *Methylobacterium* (28), *Ralstonia* (10), *Rhodopseudomonas* (21) and *Burkholderia* (91). 7/460 points lie between the interspecies region (ANIm <93% and ANIstt <94%) and the intraspecies region (ANIm >95% and ANIstt >96%). The thick and fine solid line(s) depicted the quadratic curve (the best curve to fit the data) and the individual confidence intervals (95%). The quadratic equation and R square are shown.

Genomic ANIm and the ANIg of the *SMc00019*-*truA*-*thrA* concatenation (ANIstt was used below) were calculated for 294 pairwise comparisons between strains in *Bradyrhizobium*, *Mesorhizobium*, *Sinorhizobium* and *Rhizobium* (Tables S3, S4, S5, and S6). Pearson and Spearman correlation coefficient values between these two data sets were 0.938 (*P*<0.0001) and 0.954 (*P*<0.0001), respectively. Although the linear curve produces a reasonable fit to these data (R^2^ = 0.881, F _(1, 292)_ = 2155.01, *P*<0.0001), the quadratic function explains substantially more of the variance (R^2^ = 0.966, F _(2, 291)_ = 4195.907, *P*<0.0001). Model comparison by using extra sum-of-squares suggests that the quadratic model is better than the linear (F _(1, 291)_ = 745.117, *P*<0.0001). The highest value of interspecies ANIstt was 94% and the lowest value of intraspecies ANIstt was 96%, and these values corresponded to 93% and 95% ANIm of genome sequences (Figure 2 and Tables S3, S4, S5, and S6). Therefore, there seems to be an ANI gap (≥2%) between the boundaries of inter- and intra-species comparisons for rhizobia. One exception was, however, found when comparing *R. leguminosarum* bv. *viciae* 3841 and *R. leguminosarum* bv. *trifolii* WSM1325, for which ANIm = 94.15% and ANIstt = 95.5%. These are unexpectedly low values for an intraspecific comparison. Both ANIm and ANIstt values between CIAT652 and the type strain CFN42 were lower than 92% indicating that CIAT652 should belong to a species other than *Rhizobium etli*. This is consistent with the recent finding that CIAT652 may belong to *R. phaseoli* rather than *R. etli* [22]. Species assignments of several widely studied rhizobial strains USDA110, NGR234, BTAi1, ORS278, 3841 etc. should be further examined by comparing ANI values between each strain and the type strains in the corresponding genus (Table S2).

Unfortunately, most rhizobial genome sequences available in the public database were not obtained from the type strains of the species. On the other hand, as described above, ANIstt (ANIg of *SMc00019*-*truA*-*thrA*) is a good surrogate for ANIm. Since these genes could potentially serve as excellent taxonomic markers, they were amplified from 67 type strains (Table S2) of species in four major rhizobial genera (*Bradyrhizobium*, *Sinorhizobium*, *Mesorhizobium* and *Rhizobium*) which account for around 80% of the species pool of rhizobia. Among 12 species of *Bradyrhizobium* (Table S7), 64/66 interspecies ANIstt values were below 94%; the exceptions were the *B. lablabi*/*B. jicamae* pair (ANIstt = 94.89%) and the *B. elkanii/B. pachyrhizi* pair (ANIstt = 96.59%). In *Sinorhizobium* (Table S8), 27/28 interspecies ANIstt values were below 94% and *S. kummerowiae* should be a later synonym of *S. meliloti* due to the high ANIstt (99.51%) between their type strains. In *Mesorhizobium* (Table S9), 204/210 interspecies ANIstt values were below 94%, exceptions were found in *M. tianshanense/M. tarimense* (97.72%), *M. temperatum/M. mediterraneum* (94.86%), *M. amorphae/M. septentrionale* (94.71%), *M. tianshanense/M. temperatum* (94.34%), *M. temperatum/M. tarimense* (94.34%) and *M. tarimense/M. mediterraneum*(94.12%). In *Rhizobium* (Table S10), 320/325 interspecies ANIstt values were below 94%, *R. yanglingense*, *R. loessense* and *R. gallicum* may belong to the same species due to their high ANIstt values (96.43%–97.79%), *R. indigoferae* and *R. leguminosarum* had ANIstt of 98.71%, ANIstt between *R. pisi* and *R. fabae* was 97.93%. Notably, considering the lowest boundary of intraspecies ANIstt 96% as described above, 621/629 currently known interspecies relationships were supported by this study and the remaining few species with interspecies ANIstt >96% (96.43%–99.51%) should be combined. Interestingly, these findings were perfectly supported by earlier MLSA and MALDI-TOF studies on *Rhizobium* and *Ensifer* (*Sinorhizobium*) [22]–[25]. Species assignments for those strains with whole genome sequences (Table S1) were also evaluated by calculating their ANIstt with type strains of related genera. *Bradyrhizobium japonicum* USDA 110 (ANIstt = 92.26%), *Sinorhizobium fredii* NGR234 (ANIstt = 93.86%), *Sinorhizobium medicae* WSM419 (ANIstt = 88.84%), *Mesorhizobium loti* MAFF303099 (ANIstt = 89.64%) and *Rhizobium leguminosarum* bv. *trifolii* WSM2304 (ANIstt = 93.20%) should represent species other than the names currently used. Moreover, *Bradyrhizobium* sp. BTAi1, *Bradyrhizobium* sp. ORS278, *Bradyrhizobium* sp. II CCBAU 43298, *Bradyrhizobium* sp. I (CCBAU 15615, CCBAU 15635 and CCBAU 15544), and *Sinorhizobium* sp. CCBAU 05631 did not belong to any known species (the highest ANIstt value between each strain and various type strains ranged from 81.04%–92.72%). *R. leguminosarum* bv. *trifolii* WSM1325 and *R. leguminosarum* bv. *viciae* 3841 had ANIstt of 95.1% and 95.5% with *R. leguminosarum* bv. *viciae* USDA 2370^T^, and these values lie between the inter- and intraspecies boundary of 94%–96%, implying a genetic continuum among these strains or an ongoing speciation process.

### Traditional rhizobial molecular markers for rhizobial taxonomy or phylogeny

MLSA has been widely used in rhizobial taxonomy and phylogeny. Martens et al [26] compared phylogenies of *dnaK*, *gltA*, *glnA*, *recA*, *thrC* and 16S rRNA genes within the genus *Ensifer* (*Sinorhizobium*). Rivas et al [10] and Nzoué et al [11] assessed partial sequence analysis for *atpD*, *recA*, *gyrB*, *rpoB*, *dnaK*, *glnA*, *glnII*, *gltA* and *thrC* in the genus *Bradyrhizobium*. Although the concatenation of certain housekeeping genes sometimes produced reasonable taxonomic resolution, incongruent phylogenies were reported among these genes in the same studies [10]–[11], [26]. *dnaK*, *glnA*, *gyrB*, *thrC*, *recA*, *atpD* and *rpoB* are among the 295 core genes of rhizobial genomes [13] (Table 1). Moreover, the ML gene trees for *dnaK* and *rpoB* were not significantly different from the reference species tree, according to the SH test. An intraspecific *rpoB* sequence similarity of 98.2% and an interspecific value of 97.7% were earlier suggested for bacterial species definition [27]. Nevertheless, according to the species assignments of rhizobia based on ANIm, intraspecies ANIg values of *rpoB* were 97.36%–100% whereas the highest value of interspecies ANIg was 97.7%. In the case of *dnaK*, intraspecies ANIg values were 97.32%–100% whereas the highest interspecies ANIg was 97.95%. Thus neither *rpoB* nor *dnaK* had a clear gap between inter- and intra-species ANIg values for rhizobia. Among the housekeeping genes, *recA*, *glnII* and *atpD* have been widely used as molecular markers to study rhizobial taxonomy, phylogeny, biogeography and population genetics [12], [23], [28]–[31]. In this study, with SH-test, significant incongruence (*P* = 0.017) was found between the ML tree of concatenated *recA*-*glnII-atpD* (Figure 1C) and that of *SMc00019*-*truA*-*thrA* (Figure 1B), whereas, as mentioned above, the latter tree was congruent with the reference species tree of rhizobia (Figure 1A). A notable number of incongruent branches within *Bradyrhizobium* and *Sinorhizobium* were found in Figure 1C as compared to Figure 1A and 1B. In line with these observations, significant recombination signal was found in the concatenated *recA*-*glnII-atpD* sequence (PHI test, P<0.0001) but not in *SMc00019*-*truA*-*thrA* (PHI test, P = 0.332).

### The application of SMc00019-truA-thrA in other prokaryotes

Interestingly, ANIstt also correlates well (Spearman correlation coefficient 0.835, *P*<0.0001) with the ANIm of strains in *Agrobacterium*, *Methylobacterium*, *Ralstonia*, *Rhodopseudomonas*, *Cupriavidus* and *Burkholderia*. In these genera, 160/166 intragenus comparisons belong to either the intraspecies ANI region (ANIm >95% and ANIstt >96%) or the interspecies region (ANIm <93% and ANIstt <94%). ANIstt could be used to evaluate current species assignments of related strains. For example, JMP134 [32] should not be referred to as *Cupriavidus necator* because it has low ANIm/ANIstt values (86.39%/81.74%) with the type strain N-1 [33], whereas H16 has been correctly named as *C. necator* (ANIm/ANIstt values are 95.59%/97.84% with N-1). Most strains, with published genomes, currently referred to as *Rhodopseudomonas palustris* should be subject to taxonomic re-evaluation, considering that 20/21 interstrain ANIm/ANIstt values are below 91%/94%. Likewise, among the completely sequenced genomes of *Ralstonia solanacearum*, CFBP2957, GMI1000 and PSI07 may not be considered as the same species due to their low values of ANIm (91.97%–92.73%) and ANIstt (85.96%–93.99%). ANIm/ANIstt also confirmed the interspecies relationships among *Burkholderia cenocepacia*, *B. mallei*, *B. glumae*, *B. gladioli* etc. Notably, *Burkholderia ambifaria* strains MC40_6 and AMMD have high intraspecies ANIm/ANIstt values (96.88%/98.55%), but 3 out of 4 ANIm values between MC40_6 and *B. cenocepacia* strains are above 94% and 1 out of 4 ANIm values between AMMD and *B. cenocepacia* is above 93%. In fact, among the seven points that are intermediate between the interspecific (ANIm <93% and ANIstt <94%) and intraspecific (ANIm >93% and ANIstt >94%) ANI regions (Figure 2), six points were found in these comparisons between *B. cenocepacia* and *B*. *ambifaria* strains. These findings are consistent with the view that *B. cepacia* complex bacteria (including *B. cenocepacia*, *B*. *ambifaria* etc.) may constitute a genetic continuum in which species have only relatively recently developed [34]. It is noteworthy that, as shown in Figure 2, a cluster of outliers are found around ANIstt = 85% and these data points all come from the comparisons between *Burkholderia* sp. CCGE1001/CCGE1002/CCGE1003 and other abovementioned *Burkholderia* species. The underlying mechanisms remained elusive, but it could be related to the distinct phylogenetic position of CCGE1001/CCGE1002/CCGE1003 in *Burkholderia* [34].

## Conclusions

Comparing the genomic ANIm with the ANIg of core genes that have a phylogeny congruent with the reference species tree of rhizobia allowed us identifying three housekeeping genes (*SMc00019*-*truA*-*thrA*) whose ANIg could also reflect the intraspecies and interspecies genomic ANIm among rhizobial strains. The limits of intraspecies ANIg (96%) and interspecies ANIg (94%) of *SMc00019*-*truA*-*thrA* provided a criterion for rhizobial species definition that confirmed the majority of known interspecies relationships of rhizobia and called for amalgamation of certain species and name corrections for some widely studied strains. Reconsidering species assignments of related strains is particular important, if any evolutionary conclusions are to be made at the species or sub-species level. Indeed, the micro-evolutionary mechanisms of rhizobia and pathogenic bacteria have recently received a great deal of attention by microbiologists. Therefore, *SMc00019*-*truA*-*thrA* could be utilized in taxonomy, phylogeny, population genetics and biogeography of related bacteria and in metagenomics surveys. The same type of approach could also be carried out on any prokaryote.

## Supporting Information

Table S1 **85 genomes used in this study.**
(DOC)

Table S2 **Lists of rhizobial type strains.**
(DOC)

Table S3 **Genomic ANI (low-left) versus ANI of** ***SMc00019-truA-thrA*** **(up-right) in** ***Bradyrhizobium*** **.**
(DOC)

Table S4 **Genomic ANI (low-left) versus ANI of** ***SMc00019-truA-thrA*** **(up-right) in** ***Sinorhizobium*** **.**
(DOC)

Table S5 **Genomic ANI (low-left) versus ANI of** ***SMc00019-truA-thrA*** **(up-right) in** ***Rhizobium*** **.**
(DOC)

Table S6 **Genomic ANI (low-left) versus ANI of** ***SMc00019-truA-thrA*** **(up-right) in** ***Mesorhizobium*** **.**
(DOC)

Table S7 **ANIstt values between type stains of** ***Bradyrhizobium*** **.**
(DOC)

Table S8 **ANIstt values between type stains of** ***Sinorhizobium*** **.**
(DOC)

Table S9 **ANIstt values between type stains of** ***Mesorhizobium*** **.**
(DOC)

Table S10 **ANIstt values between type stains of** ***Rhizobium*** **.**
(DOC)
