# Supplementary material for: Robust Markers Reflecting Phylogeny and Taxonomy of Rhizobia
Source: PLoS One. 2012 Sep 17;7(9):e44936. doi: 10.1371/journal.pone.0044936 (PMC3444505; doi:10.1371/journal.pone.0044936)
Supplement: Table S1 — 85 genomes used in this study. (DOC) [file pone.0044936.s001.doc]

**Table S1. 85 genomes used in this study.**

| Organism | BioProject | Accession Number* |
| --- | --- | --- |
| *Agrobacterium* | | |
| *Agrobacterium* sp. H13-3 | PRJNA50341 |  |
| *Agrobacterium radiobacter* K84 | PRJNA13402 |  |
| *Agrobacterium tumefaciens* C58 | PRJNA283 |  |
| *Agrobacterium vitis* S4 | PRJNA13372 |  |
| *Bradyrhizobium* | | |
| *Bradyrhizobium elkanii* CCBAU 05737 | PRJNA77219 | AJPV00000000 |
| *Bradyrhizobium elkanii* CCBAU 43297 | PRJNA77219 | AJPW00000000 |
| *Bradyrhizobium japonicum* CCBAU 15354 | PRJNA77219 | AJPX00000000 |
| *Bradyrhizobium japonicum* CCBAU 1551 | PRJNA77219 | AJPY00000000 |
| *Bradyrhizobium japonicum* CCBAU 15618 | PRJNA77219 | AJPZ00000000 |
| *Bradyrhizobium japonicum* CCBAU 25435 | PRJNA77219 | AJQA00000000 |
| *Bradyrhizobium japonicum* CCBAU 83623 | PRJNA77219 | AJQB00000000 |
| *Bradyrhizobium liaoningense* CCBAU 05525 | PRJNA77219 | AJQC00000000 |
| *Bradyrhizobium liaoningense* CCBAU 83689 | PRJNA77219 | AJQD00000000 |
| *Bradyrhizobium* sp. CCBAU 43298 | PRJNA77219 | AJQE00000000 |
| *Bradyrhizobium* sp. I CCBAU 15544 | PRJNA77219 | AJQF00000000 |
| *Bradyrhizobium* sp. I CCBAU 15615 | PRJNA77219 | AJQG00000000 |
| *Bradyrhizobium* sp. I CCBAU 15635 | PRJNA77219 | AJQH00000000 |
| *Bradyrhizobium* sp. CCBAU 41267 | PRJNA77219 | AJQI00000000 |
| *Bradyrhizobium yuanmingense* CCBAU 05623 | PRJNA77219 | AJQJ00000000 |
| *Bradyrhizobium yuanmingense* CCBAU 25021 | PRJNA77219 | AJQK00000000 |
| *Bradyrhizobium yuanmingense* CCBAU 35157 | PRJNA77219 | AJQL00000000 |
| *Bradyrhziobium* sp. USDA 110 | PRJNA121067 |  |
| *Bradyrhziobium* sp. BTAi1 | PRJNA16137 |  |
| *Bradyrhziobium* sp. ORS278 | PRJNA19575 |  |
| *Burkholdeira* | | |
| *Burkholderia cenocepacia* HI2424 | PRJNA13918 |  |
| *Burkholderia cenocepacia* J2315 | PRJNA339 |  |
| *Burkholderia cenocepacia* MC0-3 | PRJNA17929 |  |
| *Burkholderia cenocepacia* AU 1054 | PRJNA13919 |  |
| *Burkholderia* CCGE1001 | PRJNA37717 |  |
| *Burkholderia* CCGE1002 | PRJNA37719 |  |
| *Burkholderia* CCGE1003 | PRJNA37721 |  |
| *Burkholderia mallei* NCTC10229 | PRJNA13943 |  |
| *Burkholderia mallei* NCTC10247 | PRJNA13946 |  |
| *Burkholderia mallei* ATCC 23344 | PRJNA57725 |  |
| *Burkholderia ambifaria* MC40 6 | PRJNA58701 |  |
| *Burkholderia ambifaria* AMMD | PRJNA13490 |  |
| *Burkholderia glumae* BGR1 | PRJNA33901 |  |
| *Burkholderia gladioli* BSR3 | PRJNA64503 |  |
| *Cupriavidus* | | |
| *Cupriavidus eutropha* JMP134 | PRJNA10646 |  |
| *Cupriavidus eutropha* H16 | PRJNA158697 |  |
| *Cupriavidus necator* N-1 | PRJNA67893 |  |
| *Cupriavidus metallidurans* CH34 | PRJNA250 |  |
| *Cupriavidus taiwanensis* LMG 19424 | PRJNA15733 |  |
| *Mesorhizobium* | | |
| *Mesorhizobium ciceri* bv. *biserrulae* WSM1271 | PRJNA48991 |  |
| *Mesorhizobium loti* MAFF303099 | PRJNA18 |  |
| *Mesorhizobium opportunistum* WSM2075 | PRJNA33861 |  |
| *Methylobacterium* | | |
| *Methylobacterium extorquens* AM1 | PRJNA20 |  |
| *Methylobacterium extorquens* DM4 | PRJNA16093 |  |
| *Methylobacterium extorquens* PA1 | PRJNA18637 |  |
| *Methylobacterium chloromethanicum* CM4 | PRJNA19527 |  |
| *Methylobacterium populi* BJ001 | PRJNA19559 |  |
| *Methylobacterium nodulans* ORS2060 | PRJNA20477 |  |
| *Methylobacterium radiotolerans* JCM2831 | PRJNA18817 |  |
| *Methylobacterium* sp. 4-46 | PRJNA18809 |  |
| *Ralsotonia* | | |
| *Ralstonia solanacearum* CFBP2957 | PRJEA50685 |  |
| *Ralstonia pickettii* 12D | PRJNA18937 |  |
| *Ralstonia pickettii* 12J | PRJNA17631 |  |
| *Ralstonia solanacearum* GMI1000 | PRJNA13 |  |
| *Ralstonia solanacearum* PSI07 | PRJEA50683 |  |
| *Rhizobium* | | |
| *Rhizobium leguminosarum* bv. *trifolii* WSM1325 | PRJNA20097 |  |
| *Rhizobium leguminosarum* bv. *trifolii* WSM2304 | PRJNA20179 |  |
| *Rhizobium leguminosarum* bv. *viciae* 3841 | PRJNA344 |  |
| *Rhizobium etli* CIAT 652 | PRJNA28021 |  |
| *Rhizobium etli* CFN 42 | PRJNA13932 |  |
| *Rhodopseudomonas* | | |
| *Rhodopseudomonas palustris* TIE-1 | PRJNA20167 |  |
| *Rhodopseudomonas palustris* CGA009 | PRJNA57 |  |
| *Rhodopseudomonas palustris* BisA53 | PRJNA15751 |  |
| *Rhodopseudomonas palustris* DX 1 | PRJNA38503 |  |
| *Rhodopseudomonas palustris* BisB18 | PRJNA15750 |  |
| *Rhodopseudomonas palustris* HaA2 | PRJNA15747 |  |
| *Rhodopseudomonas palustris* BisB5 | PRJNA15749 |  |
| *Sinorhizobium* | | |
| *Sinorhizobium fredii* CCBAU 05557 | PRJNA77219 | AJQM00000000 |
| *Sinorhizobium fredii* CCBAU 25509 | PRJNA77219 | AJQN00000000 |
| *Sinorhizobium fredii* CCBAU 45436 | PRJNA77219 | AJQO00000000 |
| *Sinorhizobium fredii* CCBAU 83622 | PRJNA77219 | AJQP00000000 |
| *Sinorhizobium fredii* CCBAU 83643 | PRJNA77219 | AJQQ00000000 |
| *Sinorhizobium fredii* CCBAU 83666 | PRJNA77219 | AJQR00000000 |
| *Sinorhizobium fredii* CCBAU 83753 | PRJNA77219 | AJQS00000000 |
| *Ensifer sojae* CCBAU 05684 | PRJNA77219 | AJQT00000000 |
| *Sinorhizobium* sp. CCBAU 05631 | PRJNA77219 | AJQU00000000 |
| *Sinorhizobium* sp. NGR 234 | PRJNA21101 |  |
| *Sinorhizobium meliloti* 1021 | PRJNA19 |  |
| *Sinorhizobium meliloti* AK83 | PRJNA41993 |  |
| *Sinorhizobium meliloti* BL225C | PRJNA42477 |  |
| *Sinorhizobium meliloti* SM11 | PRJNA41117 |  |

* Accession numbers of draft genomes were shown and those of complete genomes are accessible through the BioProject number
