# Supplementary material for: Robust Markers Reflecting Phylogeny and Taxonomy of Rhizobia
Source: PLoS One. 2012 Sep 17;7(9):e44936. doi: 10.1371/journal.pone.0044936 (PMC3444505; doi:10.1371/journal.pone.0044936)
Supplement: Table S2 — Lists of rhizobial type strains. (DOC) [file pone.0044936.s002.doc]

**Table S2. Lists of rhizobial type strains.**

| Strain Name | Strain Number |
| --- | --- |
| *Bradyrhizobium* | |
| *Bradyrhizobium yuanmingense* | CCBAU 10071^T^ |
| *Bradyrhizobium japonicum* | USDA 6^T^ |
| *Bradyrhizobium daqingense* | CCBAU 15774^T^ |
| *Bradyrhizobium betae* | LMG 21987^T^ |
| *Bradyrhizobium huanghuaihaiense* | CCBAU 23303^T^ |
| *Bradyrhizobium canariense* | BTA-1^T^ |
| *Bradyrhizobium elkanii* | USDA 76^T^ |
| *Bradyrhizobium liaoningense* | USDA 3622^T^ |
| *Bradyrhizobium jicamae* | LMG 24556^T^ |
| *Bradyrhizobium lablabi* | CCBAU 23086^T^ |
| *Bradyrhizobium iriomotense* | LMG 24129^T^ |
| *Bradyrhizobium pachyrhizi* | LMG 24246^T^ |
| *Mesorhizobium* | |
| *Mesorhizobium robiniae* | CCNWYC 115^T^ |
| *Mesorhizobium caraganae* | CCBAU 11299^T^ |
| *Mesorhizobium metallidurans* | LMG 24485^T^ |
| *Mesorhizobium amorphae* | ACCC 19665^T^ |
| *Mesorhizobium chacoense* | ACCC 19665^T^ |
| *Mesorhizobium silamurunense* | CCBAU 01550^T^ |
| *Mesorhizobium huakuii* | CCBAU 02609^T^ |
| *Mesorhizobium loti* | NZP 2213^T^ |
| *Mesorhizobium camelthorni* | CCNWXJ 40-4^T^ |
| *Mesorhizobium ciceri* | USDA 3383^T^ |
| *Mesorhizobium tianshanense* | CCBAU 3306^T^ |
| *Mesorhizobium septentrionale* | SDW014^T^ |
| *Mesorhizobium plurifarium* | LMG 11892^T^ |
| *Mesorhizobium alhagi* | CCNWXJ 12-2^T^ |
| *Mesorhizobium australicum* | LMG 24608^T^ |
| *Mesorhizobium opportunistum* | LMG 24607^T^ |
| *Mesorhizobium shangrilense* | CCBAU 65327^T^ |
| *Mesorhizobium albiziae* | CCBAU 61158^T^ |
| *Mesorhizobium temperatum* | SDW018^T^ |
| *Mesorhizobium tarimense* | CCBAU 83306^T^ |
| *Mesorhizobium mediterraneum* | USDA 3392^T^ |
| *Rhizobium* | |
| *Rhizobium yanglingense* | CCBAU 71623^T^ |
| *Rhizobium tropici* A | CFN 299^T^ |
| *Rhizobium pisi* | DSM 30132^T^ |
| *Rhizobium gallicum* | KACC 10719^T^ |
| *Rhizobium selenitireducens* | LMG 24075^T^ |
| *Rhizobium alkalisoli* | CCBAU 01393^T^ |
| *Rhizobium fabae* | CCBAU 33202^T^ |
| *Rhizobium multihospitium* | CCBAU 83401^T^ |
| *Rhizobium etli* | CFN 42^T^ |
| *Rhizobium hainanense* | CCBAU 57015^T^ |
| *Rhizobium herbae* | CCBAU 83011^T^ |
| *Rhizobium mesosinicum* | CCBAU 25010^T^ |
| *Rhizobium huautlense* | SO2^T^ |
| *Rhizobium sullae* | USDA 4950^T^ |
| *Rhizobium tibeticum* | CCBAU 85039^T^ |
| *Rhizobium alamii* | LMG 24466^T^ |
| *Rhizobium daejeonense* | CCBAU 10050^T^ |
| *Rhizobium tropici* B | CIAT 899^T^ |
| *Rhizobium galegae* | HAMBI 540^T^ |
| *Rhizobium tubonense* | CCBAU 85046^T^ |
| *Rhizobium loessense* | CCBAU 7190B^T^ |
| *Rhizobium giardinii* | USDA 2914^T^ |
| *Rhizobium miluonense* | CCBAU 41251^T^ |
| *Rhizobium indigoferae* | CCBAU 71042^T^ |
| *Rhizobium vignae* | CCBAU 05106^T^ |
| *Rhizobium leguminosarum* | USDA 2370^T^ |
| *Sinorhizobium* | |
| *Sinorhizobium sojae* | CCBAU 05684^T^ |
| *Sinorhizobium medicae* | USDA 1037^T^ |
| *Sinorhizobium meliloti* | USDA 1002^T^ |
| *Sinorhizobium morelense* | Lc 04^T^ |
| *Sinorhizobium fredii* | USDA 205^T^ |
| *Sinorhizobium arboris* | HAMBI 1552^T^ |
| *Sinorhizobium kummerowiae* | CCBAU 71714^T^ |
| *Sinorhizobium americanum* | CFNEI 156^T^ |
