# Supplementary material for: Robust Markers Reflecting Phylogeny and Taxonomy of Rhizobia
Source: PLoS One. 2012 Sep 17;7(9):e44936. doi: 10.1371/journal.pone.0044936 (PMC3444505; doi:10.1371/journal.pone.0044936)
Supplement: Table S3 — Genomic ANI (low-left) versus ANI of SMc00019-truA-thrA (up-right) in Bradyrhizobium . (DOC) [file pone.0044936.s003.doc]

**Table S3. Genomic ANI (low-left) versus ANI of *SMc00019-truA-thrA* (up-right) in *Bradyrhizobium*.**

| Genome | B1 | B2 | B3 | B4 | B5 | B6 | B7 | B8 | B9 | B10 | B11 | B12 | B13 | B14 | B15 | B16 | B17 | B18 | B19 | B20 |
| --- | --- | --- | --- | --- | --- | --- | --- | --- | --- | --- | --- | --- | --- | --- | --- | --- | --- | --- | --- | --- |
| (1) *B.* sp. BTAi1 | --- | 87.44 | 79.84 | 79.45 | 79.92 | 79.92 | 79.11 | 79.11 | 79.11 | 79.65 | 79.49 | 79.69 | 79.99 | 79.84 | 79.61 | 79.61 | 79.53 | 79.72 | 79.72 | 79.65 |
| (2) *B.* sp. ORS278 | 87.91 | --- | 78.80 | 79.11 | 80.26 | 80.26 | 79.42 | 79.42 | 79.42 | 79.76 | 79.88 | 79.92 | 79.84 | 80.03 | 79.53 | 79.69 | 79.49 | 79.61 | 79.61 | 80.45 |
| (3) *B. elkanii* 43297 | 85.00 | 84.96 | --- | 97.47 | 83.18 | 83.18 | 82.10 | 82.10 | 82.10 | 82.14 | 82.22 | 83.41 | 82.57 | 83.33 | 82.18 | 82.22 | 82.10 | 83.14 | 83.14 | 82.76 |
| (4) *B. elkanii* 05737 | 84.94 | 84.93 | 95.59 | --- | 83.10 | 83.10 | 81.91 | 81.91 | 81.91 | 81.76 | 81.91 | 83.22 | 82.41 | 83.18 | 82.30 | 82.30 | 82.18 | 82.95 | 82.95 | 82.91 |
| (5) *B. liaoningense* 05525 | 84.86 | 84.85 | 86.05 | 85.66 | --- | 100 | 90.86 | 90.86 | 90.86 | 90.71 | 90.94 | 92.63 | 92.63 | 92.59 | 91.71 | 91.82 | 91.59 | 90.82 | 90.82 | 93.05 |
| (6) *B. liaoningense* 83689 | 84.85 | 84.86 | 85.92 | 85.67 | 99.53 | --- | 90.86 | 90.86 | 90.86 | 90.71 | 90.94 | 92.63 | 92.63 | 92.59 | 91.71 | 91.82 | 91.59 | 90.82 | 90.82 | 93.05 |
| (7) *B. japonicum* 15517 | 84.79 | 84.82 | 85.94 | 85.56 | 89.54 | 89.44 | --- | 100 | 100 | 96.54 | 96.70 | 90.82 | 90.59 | 90.75 | 91.24 | 91.28 | 91.21 | 91.40 | 91.40 | 90.94 |
| (8) *B. japonicum* 83623 | 84.80 | 84.81 | 86.14 | 85.60 | 89.58 | 89.43 | 99.46 | --- | 100 | 96.54 | 96.70 | 90.82 | 90.59 | 90.75 | 91.24 | 91.28 | 91.21 | 91.40 | 91.40 | 90.94 |
| (9) *B. japonicum* 15354 | 84.82 | 84.82 | 86.27 | 85.65 | 89.79 | 89.45 | 99.55 | 99.51 | --- | 96.54 | 96.70 | 90.82 | 90.59 | 90.75 | 91.24 | 91.28 | 91.21 | 91.40 | 91.40 | 90.94 |
| (10) *B. japonicum* 15618 | 84.76 | 84.79 | 85.88 | 86.42 | 89.32 | 89.17 | 95.46 | 95.46 | 95.49 | --- | 98.89 | 90.67 | 90.48 | 90.71 | 91.09 | 91.13 | 91.01 | 91.97 | 91.97 | 91.01 |
| (11) *B. japonicum* 25435 | 84.76 | 84.77 | 85.76 | 85.56 | 89.35 | 89.23 | 95.47 | 95.45 | 95.48 | 98.18 | --- | 90.90 | 90.71 | 90.90 | 91.36 | 91.40 | 91.28 | 91.94 | 91.94 | 91.05 |
| (12) *B. yuanmingense* 25021 | 84.81 | 84.82 | 85.59 | 85.48 | 89.84 | 89.87 | 88.28 | 88.29 | 88.32 | 88.21 | 88.33 | --- | 97.85 | 99.19 | 91.47 | 91.44 | 91.44 | 90.86 | 90.86 | 91.90 |
| (13) *B. yuanmingense* 05623 | 84.85 | 84.88 | 85.74 | 86.49 | 89.89 | 89.90 | 88.34 | 88.33 | 88.45 | 88.65 | 88.40 | 96.61 | --- | 97.62 | 91.24 | 91.21 | 91.13 | 90.78 | 90.78 | 91.97 |
| (14) *B. yuanmingense* 35157 | 84.82 | 84.86 | 85.35 | 85.28 | 89.79 | 89.81 | 88.23 | 88.23 | 88.25 | 88.19 | 88.35 | 97.69 | 96.92 | --- | 91.36 | 91.32 | 91.32 | 90.82 | 90.82 | 91.94 |
| (15) *B.* sp. I 15544 | 84.77 | 84.85 | 85.65 | 86.40 | 89.83 | 89.71 | 89.87 | 89.84 | 89.94 | 90.37 | 90.26 | 88.83 | 89.17 | 88.85 | --- | 99.81 | 99.62 | 91.82 | 91.82 | 91.36 |
| (16) *B.* sp. I 15635 | 84.77 | 84.85 | 85.63 | 86.41 | 89.80 | 89.72 | 89.81 | 89.80 | 89.85 | 90.28 | 90.19 | 88.80 | 89.19 | 88.86 | 98.74 | --- | 99.42 | 91.90 | 91.90 | 91.44 |
| (17) *B.* sp. I 15615 | 84.87 | 84.83 | 85.94 | 86.53 | 90.27 | 89.96 | 89.98 | 89.98 | 90.25 | 90.28 | 90.22 | 88.94 | 89.31 | 88.85 | 98.68 | 98.77 | --- | 91.74 | 91.74 | 91.28 |
| (18) *B.* sp. 41267 | 84.80 | 84.87 | 85.67 | 86.40 | 89.69 | 89.60 | 90.22 | 90.21 | 90.25 | 90.71 | 90.38 | 88.64 | 88.96 | 88.61 | 90.39 | 90.32 | 90.35 | --- | 100 | 91.44 |
| (19) *B.* sp. USDA 110 | 84.84 | 84.92 | 86.01 | 86.51 | 89.97 | 89.68 | 90.32 | 90.33 | 90.55 | 90.81 | 90.53 | 88.73 | 89.12 | 88.68 | 90.54 | 90.46 | 90.69 | 99.67 | --- | 91.44 |
| (20) *B.* sp. II 43298 | 84.78 | 84.80 | 85.66 | 86.41 | 92.00 | 91.94 | 89.49 | 89.49 | 89.54 | 89.95 | 89.82 | 89.62 | 89.94 | 89.62 | 90.62 | 90.64 | 90.79 | 90.23 | 90.37 | --- |

Note: genomic ANI values of *Bradyrhizobium* were obtained from Tian et al. (2012).

Tian CF, Zhou YJ, Zhang YM, Li QQ, Zhang YZ, et al. (2012) Comparative genomics of rhizobia nodulating soybean suggests extensive recruitment of lineage-specific genes in adaptations. Proc Natl Acad Sci U S A. (doi:10.1073/pnas.1120436109)
