# Supplementary material for: Robust Markers Reflecting Phylogeny and Taxonomy of Rhizobia
Source: PLoS One. 2012 Sep 17;7(9):e44936. doi: 10.1371/journal.pone.0044936 (PMC3444505; doi:10.1371/journal.pone.0044936)
Supplement: Table S4 — Genomic ANI (low-left) versus ANI of SMc00019-truA-thrA (up-right) in Sinorhizobium . (DOC) [file pone.0044936.s004.doc]

**Table S4. Genomic ANI (low-left) versus ANI of *SMc00019-truA-thrA* (up-right) in *Sinorhizobium*.**

| Genome | S1 | S2 | S3 | S4 | S5 | S6 | S7 | S8 | S9 | S10 | S11 | S12 | S13 | S14 |
| --- | --- | --- | --- | --- | --- | --- | --- | --- | --- | --- | --- | --- | --- | --- |
| (1) *S. fredii* CCBAU 25509 | --- | 98.35 | 98.65 | 98.65 | 100 | 98.35 | 97.72 | 90.34 | 84.94 | 93.90 | 83.00 | 83.03 | 83.00 | 83.03 |
| (2) *S. fredii* CCBAU 83753 | 98.81 | --- | 99.25 | 99.25 | 98.35 | 100 | 99.25 | 90.52 | 84.94 | 93.97 | 83.18 | 83.26 | 83.18 | 83.22 |
| (3) *S. fredii* CCBAU 45436 | 97.60 | 97.57 | --- | 100 | 98.65 | 99.25 | 98.61 | 90.64 | 84.91 | 93.82 | 83.18 | 83.22 | 83.18 | 83.22 |
| (4) *S. fredii* CCBAU 05557 | 97.53 | 97.55 | 99.65 | --- | 98.65 | 99.25 | 98.61 | 90.64 | 84.91 | 93.82 | 83.18 | 83.22 | 83.18 | 83.22 |
| (5) *S. fredii* CCBAU 83622 | 99.09 | 98.25 | 97.59 | 97.61 | --- | 98.35 | 97.72 | 90.34 | 84.94 | 93.90 | 83.00 | 83.03 | 83.00 | 83.03 |
| (6) *S. fredii* CCBAU 83643 | 97.56 | 98.41 | 97.27 | 97.19 | 97.59 | --- | 99.25 | 90.52 | 84.94 | 93.97 | 83.18 | 83.26 | 83.18 | 83.22 |
| (7) *S. fredii* CCBAU 83666 | 98.12 | 98.73 | 97.28 | 97.24 | 98.61 | 98.08 | --- | 90.49 | 85.13 | 93.86 | 83.22 | 83.30 | 83.22 | 83.26 |
| (8) *S.* sp. CCBAU 05631 | 89.35 | 89.17 | 89.29 | 89.18 | 89.13 | 89.09 | 89.59 | --- | 84.16 | 90.97 | 83.56 | 83.63 | 83.60 | 83.60 |
| (9) *S. sojae* CCBAU 05684 | 87.53 | 87.43 | 87.61 | 87.39 | 87.56 | 87.34 | 86.89 | 86.69 | --- | 85.02 | 83.63 | 83.71 | 83.71 | 83.67 |
| (10) *S.* sp. NGR234 | 92.31 | 92.24 | 92.44 | 92.39 | 92.36 | 92.25 | 92.31 | 89.19 | 86.72 | --- | 83.67 | 83.75 | 83.67 | 83.71 |
| (11) *S. meliloti* 1021 | 85.73 | 85.76 | 85.81 | 85.71 | 85.72 | 85.68 | 85.76 | 85.66 | 85.33 | 85.76 | --- | 99.81 | 99.93 | 99.96 |
| (12) *S. meliloti* AK83 | 85.76 | 85.84 | 85.95 | 85.81 | 85.71 | 85.82 | 85.76 | 85.77 | 85.40 | 85.82 | 99.10 | --- | 99.81 | 99.85 |
| (13) *S. meliloti* BL225C | 85.76 | 85.86 | 85.89 | 85.72 | 85.75 | 85.80 | 85.81 | 85.69 | 85.39 | 85.76 | 99.38 | 98.96 | --- | 99.96 |
| (14) *S. meliloti* SM11 | 85.76 | 85.83 | 85.91 | 85.76 | 85.74 | 85.77 | 85.77 | 85.76 | 85.41 | 85.77 | 99.19 | 98.97 | 99.05 | --- |
