# Supplementary material for: Robust Markers Reflecting Phylogeny and Taxonomy of Rhizobia
Source: PLoS One. 2012 Sep 17;7(9):e44936. doi: 10.1371/journal.pone.0044936 (PMC3444505; doi:10.1371/journal.pone.0044936)
Supplement: Table S5 — Genomic ANI (low-left) versus ANI of SMc00019-truA-thrA (up-right) in Rhizobium . (DOC) [file pone.0044936.s005.doc]

**Table S5. Genomic ANI (low-left) versus ANI of *SMc00019-truA-thrA* (up-right) in *Rhizobium*.**

| Genome | R1 | R2 | R3 | R4 | R5 |
| --- | --- | --- | --- | --- | --- |
| (1) *R. leguminosarum* bv. *trifolii* WSM1325 | --- | 92.54 | 95.5 | 91.22 | 90.78 |
| (2) *R. leguminosarum* bv. *trifolii* WSM2304 | 89.26 | --- | 92.94 | 91.66 | 91.4 |
| (3) *R. leguminosarum* bv. *viciae* 3841 | 94.15 | 89.35 | --- | 91.26 | 90.63 |
| (4) *R. etli* CFN 652 | 88.23 | 88.97 | 88.26 | --- | 93.34 |
| (5) *R. etli* CFN 42 | 87.82 | 88.36 | 87.89 | 90.43 | --- |
