# Supplementary material for: Robust Markers Reflecting Phylogeny and Taxonomy of Rhizobia
Source: PLoS One. 2012 Sep 17;7(9):e44936. doi: 10.1371/journal.pone.0044936 (PMC3444505; doi:10.1371/journal.pone.0044936)
Supplement: Table S6 — Genomic ANI (low-left) versus ANI of SMc00019-truA-thrA (up-right) in Mesorhizobium . (DOC) [file pone.0044936.s006.doc]

**Table S6. Genomic ANI (low-left) versus ANI of *SMc00019-truA-thrA* (up-right) in *Mesorhizobium*.**

| Genome | M1 | M2 | M3 |
| --- | --- | --- | --- |
| (1) *M. ciceri* bv. *biserrulae* WSM1271 | --- | 89.78 | 90.37 |
| (2) *M. loti* MAFF303099 | 87.82 | --- | 91.39 |
| (3) *M. opportunistum* WSM2075 | 89.34 | 89.12 | --- |
