# Supplementary material for: Robust Markers Reflecting Phylogeny and Taxonomy of Rhizobia
Source: PLoS One. 2012 Sep 17;7(9):e44936. doi: 10.1371/journal.pone.0044936 (PMC3444505; doi:10.1371/journal.pone.0044936)
Supplement: Table S7 — ANIstt values between type stains of Bradyrhizobium . (DOC) [file pone.0044936.s007.doc]

**Table S7. ANIstt values between type stains of *Bradyrhizobium*.**

|  | B1 | B2 | B3 | B4 | B5 | B6 | B7 | B8 | B9 | B10 | B11 | B12 |
| --- | --- | --- | --- | --- | --- | --- | --- | --- | --- | --- | --- | --- |
| (1) *B. yuanmingense* |  |  |  |  |  |  |  |  |  |  |  |  |
| (2) *B. japonicum* | 91.41 |  |  |  |  |  |  |  |  |  |  |  |
| (3) *B. daqingense* | 91.56 | 90.56 |  |  |  |  |  |  |  |  |  |  |
| (4) *B. betae* | 92.41 | 92.65 | 90.71 |  |  |  |  |  |  |  |  |  |
| (5) *B. huanghuaihaiense* | 92.34 | 91.56 | 90.71 | 92.72 |  |  |  |  |  |  |  |  |
| (6) *B. canariense* | 90.94 | 92.72 | 90.56 | 92.34 | 91.41 |  |  |  |  |  |  |  |
| (7) *B. elkanii* | 84.13 | 82.51 | 82.89 | 83.20 | 83.90 | 83.75 |  |  |  |  |  |  |
| (8) *B. liaoningense* | 93.50 | 90.63 | 90.63 | 91.18 | 91.25 | 91.10 | 84.44 |  |  |  |  |  |
| (9) *B. jicamae* | 82.89 | 83.05 | 82.12 | 82.28 | 82.66 | 82.97 | 84.52 | 83.28 |  |  |  |  |
| (10) *B. lablabi* | 83.67 | 83.36 | 82.74 | 83.13 | 82.66 | 83.28 | 85.06 | 83.44 | 94.89 |  |  |  |
| (11) *B. iriomotense* | 89.01 | 88.93 | 88.78 | 90.71 | 89.40 | 89.40 | 82.74 | 89.32 | 82.89 | 82.74 |  |  |
| (12) *B. pachyrhizi* | 84.37 | 82.97 | 83.05 | 83.67 | 84.29 | 83.82 | 96.59 | 84.75 | 84.06 | 84.91 | 83.28 |  |
