# Supplementary material for: Robust Markers Reflecting Phylogeny and Taxonomy of Rhizobia
Source: PLoS One. 2012 Sep 17;7(9):e44936. doi: 10.1371/journal.pone.0044936 (PMC3444505; doi:10.1371/journal.pone.0044936)
Supplement: Table S8 — ANIstt values between type stains of Sinorhizobium . (DOC) [file pone.0044936.s008.doc]

**Table S8. ANIstt values between type stains of *Sinorhizobium*.**

|  | S1 | S2 | S3 | S4 | S5 | S6 | S7 | S8 |
| --- | --- | --- | --- | --- | --- | --- | --- | --- |
| (1) *S. sojae* |  |  |  |  |  |  |  |  |
| (2) *S. medicae* | 81.50 |  |  |  |  |  |  |  |
| (3) *S. meliloti* | 84.60 | 84.46 |  |  |  |  |  |  |
| (4) *S. morelense* | 82.13 | 79.87 | 81.85 |  |  |  |  |  |
| (5) *S. fredii* | 85.66 | 80.93 | 83.33 | 80.23 |  |  |  |  |
| (6) *S. arboris* | 84.60 | 85.10 | 93.22 | 81.43 | 83.76 |  |  |  |
| (7) *S. kummerowiae* | 84.75 | 84.60 | 99.51 | 81.92 | 83.40 | 92.94 |  |  |
| (8) *S. americanum* | 84.60 | 81.36 | 84.11 | 80.30 | 89.83 | 83.76 | 84.04 |  |
