# Supplementary material for: Robust Markers Reflecting Phylogeny and Taxonomy of Rhizobia
Source: PLoS One. 2012 Sep 17;7(9):e44936. doi: 10.1371/journal.pone.0044936 (PMC3444505; doi:10.1371/journal.pone.0044936)
Supplement: Table S9 — ANIstt values between type stains of Mesorhizobium . (DOC) [file pone.0044936.s009.doc]

**Table S9. ANIstt values between type stains of *Mesorhizobium*.**

|  | M1 | M2 | M3 | M4 | M5 | M6 | M7 | M8 | M9 | M10 | M11 | M12 | M13 | M14 | M15 | M16 | M17 | M18 | M19 | M20 | M21 |
| --- | --- | --- | --- | --- | --- | --- | --- | --- | --- | --- | --- | --- | --- | --- | --- | --- | --- | --- | --- | --- | --- |
| (1) *M. robiniae* |  |  |  |  |  |  |  |  |  |  |  |  |  |  |  |  |  |  |  |  |  |
| (2) *M. caraganae* | 87.51 |  |  |  |  |  |  |  |  |  |  |  |  |  |  |  |  |  |  |  |  |
| (3) *M. metallidurans* | 89.35 | 88.61 |  |  |  |  |  |  |  |  |  |  |  |  |  |  |  |  |  |  |  |
| (4) *M. amorphae* | 87.29 | 88.76 | 88.54 |  |  |  |  |  |  |  |  |  |  |  |  |  |  |  |  |  |  |
| (5) *M. chacoense* | 79.50 | 79.13 | 78.25 | 79.21 |  |  |  |  |  |  |  |  |  |  |  |  |  |  |  |  |  |
| (6) *M. silamurunense* | 86.41 | 87.95 | 87.58 | 88.76 | 78.55 |  |  |  |  |  |  |  |  |  |  |  |  |  |  |  |  |
| (7) *M. huakuii* | 87.36 | 89.05 | 88.32 | 88.46 | 79.72 | 88.91 |  |  |  |  |  |  |  |  |  |  |  |  |  |  |  |
| (8) *M. loti* | 88.54 | 88.54 | 88.10 | 87.66 | 78.62 | 87.29 | 91.55 |  |  |  |  |  |  |  |  |  |  |  |  |  |  |
| (9) *M. camelthorni* | 79.50 | 81.93 | 80.97 | 82.07 | 78.18 | 80.31 | 81.85 | 80.75 |  |  |  |  |  |  |  |  |  |  |  |  |  |
| (10) *M. ciceri* | 87.95 | 88.10 | 87.44 | 87.07 | 78.18 | 87.14 | 90.45 | 92.58 | 79.87 |  |  |  |  |  |  |  |  |  |  |  |  |
| (11) *M. tianshanense* | 93.68 | 86.99 | 89.49 | 87.14 | 79.13 | 86.33 | 87.14 | 87.22 | 79.94 | 86.99 |  |  |  |  |  |  |  |  |  |  |  |
| (12) *M. septentrionale* | 88.46 | 88.61 | 89.42 | 94.71 | 78.77 | 89.20 | 88.76 | 88.46 | 81.34 | 87.66 | 87.95 |  |  |  |  |  |  |  |  |  |  |
| (13) *M. plurifarium* | 85.67 | 86.85 | 86.48 | 87.22 | 78.77 | 89.27 | 87.14 | 86.11 | 80.09 | 86.33 | 84.50 | 87.58 |  |  |  |  |  |  |  |  |  |
| (14) *M. alhagi* | 79.79 | 82.00 | 81.70 | 81.56 | 78.10 | 79.72 | 81.78 | 80.90 | 92.95 | 80.09 | 79.50 | 81.12 | 80.01 |  |  |  |  |  |  |  |  |
| (15) *M. australicum* | 85.82 | 87.88 | 87.07 | 87.36 | 78.47 | 86.85 | 91.18 | 89.93 | 80.24 | 88.68 | 84.35 | 87.66 | 85.67 | 80.16 |  |  |  |  |  |  |  |
| (16) *M. opportunistum* | 88.24 | 88.68 | 87.95 | 89.57 | 80.38 | 88.32 | 92.36 | 91.26 | 81.63 | 90.15 | 87.36 | 90.23 | 86.70 | 81.41 | 91.11 |  |  |  |  |  |  |
| (17) *M. shangrilense* | 86.77 | 87.07 | 85.75 | 86.11 | 77.74 | 86.11 | 87.07 | 88.24 | 80.46 | 86.70 | 85.89 | 86.63 | 85.01 | 80.53 | 85.75 | 86.48 |  |  |  |  |  |
| (18) *M. albiziae* | 81.04 | 82.29 | 79.43 | 80.90 | 84.86 | 80.90 | 80.75 | 79.94 | 80.01 | 79.50 | 80.46 | 79.94 | 80.31 | 79.57 | 80.31 | 80.75 | 79.94 |  |  |  |  |
| (19) *M. temperatum* | 93.61 | 87.73 | 89.86 | 87.95 | 79.35 | 87.14 | 87.73 | 88.10 | 80.90 | 87.66 | 94.34 | 88.46 | 85.38 | 80.68 | 86.19 | 88.10 | 86.77 | 81.04 |  |  |  |
| (20) *M. tarimense* | 93.61 | 87.29 | 89.64 | 87.22 | 78.84 | 86.26 | 86.99 | 87.44 | 80.09 | 86.85 | 97.72 | 88.10 | 84.57 | 79.72 | 84.42 | 87.36 | 86.11 | 80.53 | 94.34 |  |  |
| (21) *M. mediterraneum* | 93.75 | 87.73 | 89.35 | 87.29 | 78.84 | 86.85 | 87.88 | 87.58 | 80.24 | 87.22 | 94.86 | 88.39 | 85.01 | 80.24 | 85.45 | 87.36 | 85.89 | 80.75 | 94.86 | 94.12 |  |
