# Supplementary material for: Robust Markers Reflecting Phylogeny and Taxonomy of Rhizobia
Source: PLoS One. 2012 Sep 17;7(9):e44936. doi: 10.1371/journal.pone.0044936 (PMC3444505; doi:10.1371/journal.pone.0044936)
Supplement: Table S10 — ANIstt values between type stains of Rhizobium . (DOC) [file pone.0044936.s010.doc]

**Table S10. ANIstt values between type stains of *Rhizobium*.**

|  | R1 | R2 | R3 | R4 | R5 | R6 | R7 | R8 | R9 | R10 | R11 | R12 | R13 | R14 | R15 | R16 | R17 | R18 | R19 | R20 | R21 | R22 | R23 | R24 | R25 | R26 |
| --- | --- | --- | --- | --- | --- | --- | --- | --- | --- | --- | --- | --- | --- | --- | --- | --- | --- | --- | --- | --- | --- | --- | --- | --- | --- | --- |
| (1) *R. yanglingense* |  |  |  |  |  |  |  |  |  |  |  |  |  |  |  |  |  |  |  |  |  |  |  |  |  |  |
| (2) *R. tropici* A | 81.50 |  |  |  |  |  |  |  |  |  |  |  |  |  |  |  |  |  |  |  |  |  |  |  |  |  |
| (3) *R. pisi* | 83.36 | 81.00 |  |  |  |  |  |  |  |  |  |  |  |  |  |  |  |  |  |  |  |  |  |  |  |  |
| (4) *R. gallicum* | 97.79 | 81.50 | 83.21 |  |  |  |  |  |  |  |  |  |  |  |  |  |  |  |  |  |  |  |  |  |  |  |
| (5) *R. selenitireducens* | 77.79 | 78.71 | 78.21 | 77.29 |  |  |  |  |  |  |  |  |  |  |  |  |  |  |  |  |  |  |  |  |  |  |
| (6) *R. alkalisoli* | 74.93 | 75.57 | 76.79 | 74.50 | 76.50 |  |  |  |  |  |  |  |  |  |  |  |  |  |  |  |  |  |  |  |  |  |
| (7) *R. fabae* | 83.50 | 80.93 | 97.93 | 83.29 | 78.36 | 77.21 |  |  |  |  |  |  |  |  |  |  |  |  |  |  |  |  |  |  |  |  |
| (8) *R. multihospitium* | 78.00 | 81.93 | 78.07 | 78.21 | 76.36 | 74.64 | 78.71 |  |  |  |  |  |  |  |  |  |  |  |  |  |  |  |  |  |  |  |
| (9) *R. etli* | 83.71 | 82.71 | 90.43 | 83.71 | 78.21 | 76.71 | 90.79 | 80.14 |  |  |  |  |  |  |  |  |  |  |  |  |  |  |  |  |  |  |
| (10) *R.* *hainanense* | 81.64 | 87.07 | 82.21 | 82.21 | 79.93 | 76.21 | 82.29 | 84.79 | 83.64 |  |  |  |  |  |  |  |  |  |  |  |  |  |  |  |  |  |
| (11) *R. herbae* | 77.00 | 76.50 | 76.29 | 76.64 | 77.93 | 74.07 | 76.43 | 75.14 | 77.00 | 76.71 |  |  |  |  |  |  |  |  |  |  |  |  |  |  |  |  |
| (12) *R. mesosinicum* | 83.57 | 80.93 | 85.71 | 83.36 | 77.21 | 75.71 | 86.00 | 77.57 | 85.29 | 81.79 | 76.14 |  |  |  |  |  |  |  |  |  |  |  |  |  |  |  |
| (13) *R. huautlense* | 73.93 | 74.86 | 76.57 | 73.79 | 76.50 | 89.36 | 76.71 | 73.50 | 75.79 | 75.79 | 73.50 | 74.29 |  |  |  |  |  |  |  |  |  |  |  |  |  |  |
| (14) *R. sullae* | 89.93 | 81.43 | 84.50 | 89.21 | 78.14 | 75.93 | 84.29 | 78.50 | 84.57 | 82.86 | 77.50 | 84.50 | 75.29 |  |  |  |  |  |  |  |  |  |  |  |  |  |
| (15) *R.* *tibeticum* | 81.93 | 80.79 | 81.07 | 82.07 | 76.29 | 74.29 | 80.79 | 77.43 | 82.43 | 81.64 | 75.71 | 82.07 | 73.79 | 82.07 |  |  |  |  |  |  |  |  |  |  |  |  |
| (16) *R.* *alamii* | 83.64 | 81.14 | 86.14 | 83.36 | 77.93 | 76.00 | 86.00 | 77.93 | 85.71 | 82.71 | 76.36 | 93.79 | 75.43 | 85.14 | 82.43 |  |  |  |  |  |  |  |  |  |  |  |
| (17) *R.* *daejeonense* | 75.29 | 76.79 | 76.00 | 75.43 | 80.36 | 74.79 | 75.71 | 73.57 | 75.29 | 76.36 | 74.29 | 75.00 | 74.57 | 76.21 | 74.36 | 75.64 |  |  |  |  |  |  |  |  |  |  |
| (18) *R. tropici* B | 81.71 | 85.86 | 81.21 | 81.57 | 78.79 | 76.71 | 81.57 | 85.93 | 83.07 | 90.64 | 76.71 | 82.00 | 75.36 | 81.43 | 80.07 | 81.86 | 76.14 |  |  |  |  |  |  |  |  |  |
| (19) *R.* *galegae* | 75.50 | 76.29 | 75.79 | 75.93 | 76.64 | 78.57 | 76.14 | 74.71 | 76.86 | 77.50 | 77.07 | 76.07 | 78.00 | 76.07 | 74.93 | 76.29 | 74.79 | 77.43 |  |  |  |  |  |  |  |  |
| (20) *R. tubonense* | 78.64 | 80.00 | 79.64 | 78.64 | 77.64 | 73.93 | 79.93 | 76.79 | 81.14 | 80.71 | 75.93 | 79.50 | 74.50 | 79.64 | 77.93 | 80.07 | 74.43 | 81.14 | 75.43 |  |  |  |  |  |  |  |
| (21) *R.* *loessense* | 97.21 | 81.14 | 83.07 | 96.43 | 77.36 | 75.07 | 83.43 | 77.71 | 83.29 | 81.29 | 77.29 | 83.21 | 74.07 | 89.86 | 81.36 | 83.36 | 75.43 | 81.00 | 75.43 | 78.64 |  |  |  |  |  |  |
| (22) *R. giardinii* | 77.57 | 78.57 | 77.57 | 77.57 | 79.07 | 73.86 | 77.36 | 74.64 | 78.29 | 84.07 | 81.43 | 77.50 | 73.93 | 78.79 | 77.57 | 78.50 | 76.00 | 80.93 | 75.71 | 77.29 | 77.86 |  |  |  |  |  |
| (23) *R*. *miluonense* | 81.00 | 87.57 | 82.21 | 81.43 | 78.50 | 76.00 | 82.07 | 83.43 | 83.07 | 89.50 | 76.21 | 82.71 | 75.36 | 81.64 | 81.57 | 82.71 | 76.29 | 88.21 | 76.14 | 79.93 | 80.50 | 79.36 |  |  |  |  |
| (24) *R.* *indigoferae* | 84.36 | 82.14 | 91.50 | 84.21 | 79.07 | 76.64 | 91.50 | 79.14 | 90.36 | 83.29 | 78.86 | 85.00 | 76.64 | 85.64 | 82.64 | 85.14 | 75.79 | 83.21 | 77.57 | 80.36 | 84.14 | 78.07 | 82.79 |  |  |  |
| (25) *R*. *vignae* | 75.21 | 76.00 | 75.86 | 76.07 | 77.43 | 78.93 | 76.21 | 74.50 | 76.86 | 77.93 | 77.50 | 76.79 | 79.14 | 76.00 | 75.00 | 76.71 | 75.14 | 77.07 | 92.43 | 75.21 | 75.43 | 76.36 | 77.00 | 77.79 |  |  |
| (26) *R*. *leguminosarum* | 84.21 | 82.36 | 92.21 | 84.29 | 79.29 | 76.64 | 92.14 | 79.50 | 90.93 | 84.14 | 78.57 | 85.79 | 77.00 | 85.86 | 83.07 | 85.79 | 75.71 | 83.79 | 77.57 | 81.07 | 84.21 | 78.50 | 83.29 | 98.71 | 78.29 |  |
